# Supplementary figures and images for: Presenilin/γ-secretase-dependent EphA3 processing mediates axon elongation through non-muscle myosin IIA
Source: eLife. 2019 Oct 2;8:e43646. doi: 10.7554/eLife.43646 (PMC6774734; doi:10.7554/eLife.43646)

# Supplementary file 1. Proteomic analysis of the PS/ $\gamma$ -secretase-mediated EphA3 cleavage site

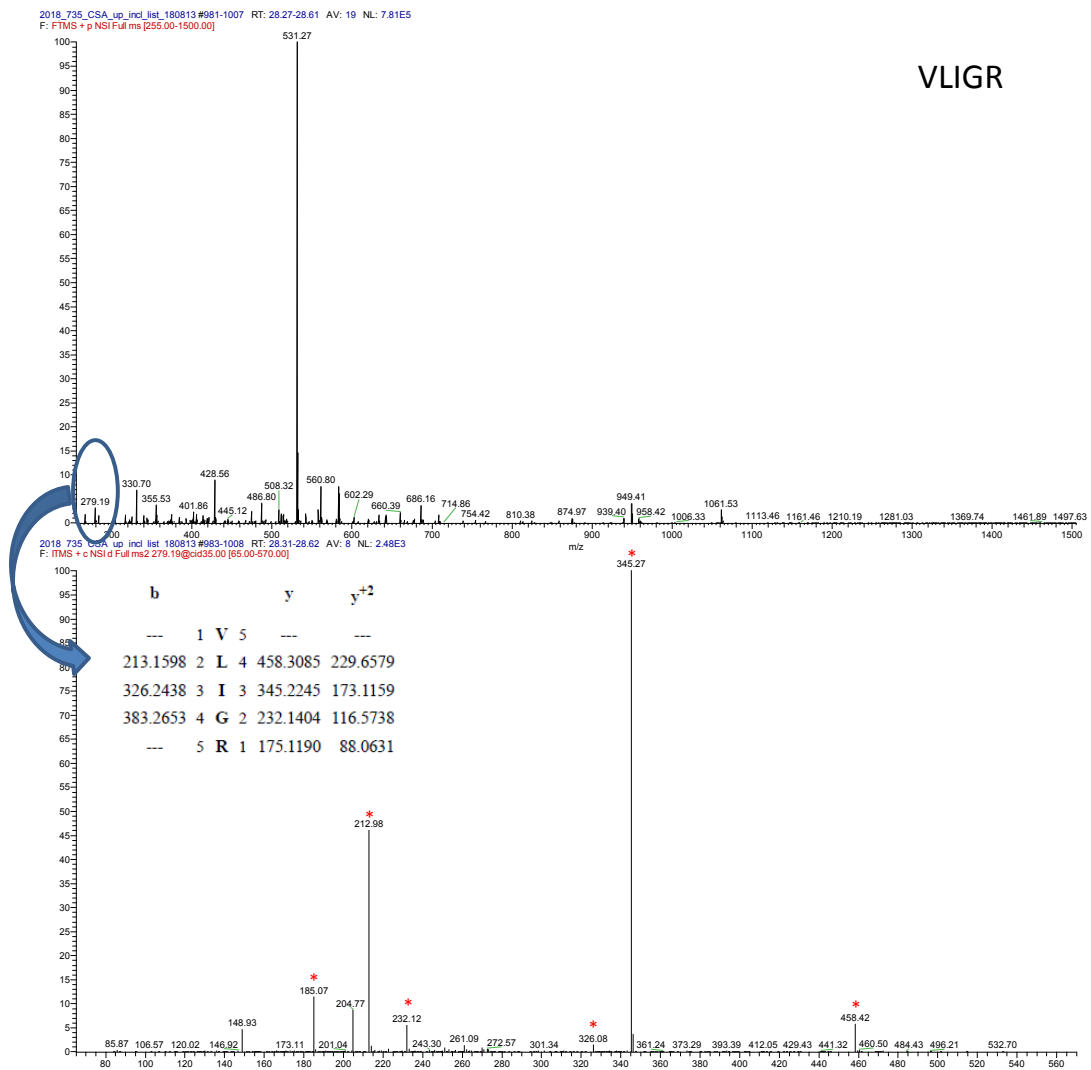

Supplement: Supplementary file 1. — Mass spectrometry spectra of the trypsin-digested band (~47–49 kDa) (top spectra) and specific mass spectrometry spectra obtained for peptide VLIGR showing the mass/charge (m/z) values (bottom spectra). Detected signals corresponding with theoretical ions are labeled with red asterisks. [file elife-43646-supp1.pdf]
