# Supplementary material for: Presenilin/γ-secretase-dependent EphA3 processing mediates axon elongation through non-muscle myosin IIA
Source: eLife. 2019 Oct 2;8:e43646. doi: 10.7554/eLife.43646 (PMC6774734; doi:10.7554/eLife.43646)
Supplement: Supplementary file 2. [file elife-43646-supp2.docx]

***Supplementary file 2*.** **Identified peptides from NMIIA protein**

| Start - End | Sequence | Molecular Mass  (Observed) |  | Error (ppm*) |
| --- | --- | --- | --- | --- |
| 144 - 165 | HEMPPHIYAITDTAYRSMMQDR | 2663.2180 |  | 0.63 |
| 187 - 199 | VIQYLAYVASSHK | 1478.7799 |  | -13.7 |
| 273 – 289 | TFHIFYYLLSGAGEHLK | 1996.0469 |  | 7.13 |
| 290 -301 | TDLLLEPYNKYR | 1524.7963 |  | -6.13 |
| 328 - 341 | IMGIPEEEQMGLLR | 1615.8153 |  | -1.76 |
| 374 – 387 | VSHLLGINVTDFTR | 1571.8421 |  | -7.58 |
| 541 - 555 | SFVEKVMQEQGTHPK | 1744.9110 |  | 24.3 |
| 566 - 580 | ADFCIIHYAGKVDYK | 1799.9290 |  | 28.1 |
| 618 – 637 | IIGLDQVAGMSETALPGAFK | 2034.0428 |  | -7.26 |
| 663 -678 | NTNPNFVRCIIPNHEK | 1953.0276 |  | 26.5 |
| 746 – 755 | ALELDSNLYR | 1193.6135 |  | -2.10 |
| 1175 - 1181 | TLEEEAK | 819.4100 |  | 0.72 |
| 1393 - 1404 | DLEGLSQRHEEK | 1440.7017 |  | -4.15 |
| 1755 – 1770 | ANLQIDQINTDLNLER | 1869.9816 |  | 8.09 |
| 1816 – 1830 | IAQLEEQLDNETKER | 1815.9179 |  | 5-31 |
| 1899 – 1912 | ELEDATETADAMNR | 1565.7169 |  | 26.9 |
| 1924 – 1933 | GDLPFVVPRR | 1155.6697 |  | 5.54 |

*ppm is the deviation of the measured mass from the theoretical mass of the peptid
