# Supplementary material for: Presenilin/γ-secretase-dependent EphA3 processing mediates axon elongation through non-muscle myosin IIA
Source: eLife. 2019 Oct 2;8:e43646. doi: 10.7554/eLife.43646 (PMC6774734; doi:10.7554/eLife.43646)
Supplement: Transparent reporting form [file elife-43646-transrepform.pdf]

eLIFE
1st Floor
24 Hills Road
Cambridge CB2 1JP, UK
P 01223 855340
W elifesciences.org
T @elife

eLife's transparent reporting form

We encourage authors to provide detailed information *within their submission* to facilitate the interpretation and replication of experiments. Authors can upload supporting documentation to indicate the use of appropriate reporting guidelines for health-related research (see EQUATOR Network), life science research (see the BioSharing Information Resource), or the ARRIVE guidelines for reporting work involving animal research. Where applicable, authors should refer to any relevant reporting standards documents in this form.

If you have any questions, please consult our Journal Policies and/or contact us: editorial@elifesciences.org.

Sample-size estimation

- You should state whether an appropriate sample size was computed when the study was being designed
- You should state the statistical method of sample size computation and any required assumptions
- If no explicit power analysis was used, you should describe how you decided what sample (replicate) size (number) to use

Please outline where this information can be found within the submission (e.g., sections or figure legends), or explain why this information doesn't apply to your submission:

All the information relative to sample size is included in the figure legends of each figure and in the Methods section (*Statistical analysis*).

Replicates

- You should report how often each experiment was performed
- You should include a definition of biological versus technical replication
- The data obtained should be provided and sufficient information should be provided to indicate the number of independent biological and/or technical replicates
- If you encountered any outliers, you should describe how these were handled
- Criteria for exclusion/inclusion of data should be clearly stated
- High-throughput sequence data should be uploaded before submission, with a private link for reviewers provided (these are available from both GEO and ArrayExpress)

Please outline where this information can be found within the submission (e.g., sections or figure legends), or explain why this information doesn't apply to your submission:

Information relative to number of replicates per experiment is included in the legend of each figure. The number of biological experiments and replicates for each experiment is specified in the Source data table. The information about treatment of outliers is specified at the Methods section (*Statistical analysis*).

eLife Sciences Publications, Ltd is a limited liability non-profit non-stock corporation incorporated in the State of Delaware, USA, with company number 5030732, and is registered in the UK with company number FC030576 and

Statistical reporting

- Statistical analysis methods should be described and justified
- Raw data should be presented in figures whenever informative to do so (typically when N per group is less than 10)
- For each experiment, you should identify the statistical tests used, exact values of N, definitions of center, methods of multiple test correction, and dispersion and precision measures (e.g., mean, median, SD, SEM, confidence intervals; and, for the major substantive results, a measure of effect size (e.g., Pearson's r, Cohen's d)
- Report exact p-values wherever possible alongside the summary statistics and 95% confidence intervals. These should be reported for all key questions and not only when the p-value is less than 0.05.

Please outline where this information can be found within the submission (e.g., sections or figure legends), or explain why this information doesn't apply to your submission:

All statistical information is included in the legend of each figure and summarized in the table 'Source Data Table'

(For large datasets, or papers with a very large number of statistical tests, you may upload a single table file with tests, Ns, etc., with reference to sections in the manuscript.)

Group allocation

- Indicate how samples were allocated into experimental groups (in the case of clinical studies, please specify allocation to treatment method); if randomization was used, please also state if restricted randomization was applied
- Indicate if masking was used during group allocation, data collection and/or data analysis

Please outline where this information can be found within the submission (e.g., sections or figure legends), or explain why this information doesn't apply to your submission:

This information doesn't apply to our submission since the experimental groups in the presented data are *in vitro* cultures or in the case of mice samples, the groups were done according to the genotype.

Additional data files ("source data")

- We encourage you to upload relevant additional data files, such as numerical data that are represented as a graph in a figure, or as a summary table
- Where provided, these should be in the most useful format, and they can be uploaded as "Source data" files linked to a main figure or table
- Include model definition files including the full list of parameters used
- Include code used for data analysis (e.g., R, MatLab)
- Avoid stating that data files are "available upon request"

Please indicate the figures or tables for which source data files have been provided:

We have included an additional table named 'Source Data Table' at the end of this document that summarizes all the information related to Statistical analyses that have been performed in this study.

eLife Sciences Publications, Ltd is a limited liability non-profit non-stock corporation incorporated in the State of Delaware, USA, with company number 5030732, and is registered in the UK with company number FC030576 and

eLIFE
1st Floor
24 Hills Road
Cambridge CB2 1JP, UK
P 01223 855340
W elifesciences.org
T @elife

Source Data Table.

Fig.	N	Statistical test	*P* value
1A	N = 3-4 animals; 10-15 section/animal; 5 axons/section	Unpaired two-tailed *t* Student	*$P < 0.05$
1B	N = 3-4 animals / genotype	Unpaired two-tailed *t* Student	*$P < 0.05$
1D	N = 3-7 experiments; 25-30 neurons/condition/experiment	One-way ANOVA; Bonferroni *post hoc* test	*$P < 0.05$
2A	N = 4 brain lysates / genotype	Unpaired two-tailed *t* Student	**$P < 0.01$
2E	N = 5 experiments	One-way ANOVA; Bonferroni *post hoc* test	*$P < 0.05$; **$P < 0.01$
2F	N = 3-5 experiments	One-way ANOVA; Bonferroni *post hoc* test	*$P < 0.05$
2G	N = 3-5 experiments (HEK293T)<br>N=1 (Brain homogenate)	One-way ANOVA; Bonferroni *post hoc* test<br>(HEK 293T)	*$P < 0.05$; ***$P < 0.001$<br>(HEK 293T)
3A	N = 3 experiments; 3-6 coverslips/experiment, 30 neurons/coverslip	Two-way ANOVA; Bonferroni *post hoc* test	*$P < 0.05$
3B	N = 3 experiments; 3-6 coverslips/experiment, 30 neurons/coverslip	Two-way ANOVA; Bonferroni *post hoc* test	*$P < 0.05$
3C	N = 3 experiments; 3-6 coverslips/experiment, 30 neurons/coverslip	Two-way ANOVA; Bonferroni *post hoc* test	*$P < 0.05$
3D	N = 3-9 sections	Unpaired two-tailed *t* Student	**$P < 0.0001$

4A	N = 3-5 experiments	Two-way ANOVA; Bonferroni *post hoc* test	$^{**}P < 0.01$
4B	N = 3 experiments; 30-40 cells per condition/experiment	Two-way ANOVA; Bonferroni *post hoc* test	$^{*}P < 0.05$
4D	N = 3 experiments; 30-40 cells per condition/experiment	One-way ANOVA; Bonferroni *post hoc* test	$^{*}P < 0.05$
5A	N = 3 experiments; 3-6 coverslips/experiment, 30 neurons/coverslip	Two-way ANOVA; Bonferroni *post hoc* test	$^{*}P < 0.05$
5B	N = 3 experiments; 3-6 coverslips/experiment, 30 neurons/coverslip	Two-way ANOVA; Bonferroni *post hoc* test	$^{*}P < 0.05$
5C	N = 3 experiments; 3-6 coverslips/experiment, 30 neurons/coverslip	Two-way ANOVA; Bonferroni *post hoc* test	$^{*}P < 0.05$
5D	N = 3 experiments; 40-80 neurons /group	Two-way ANOVA; Bonferroni *post hoc* test	$^{*}P < 0.05$; $^{**}P < 0.01$; ****P < 0.0001
5E	N = 3-4 experiments	Two-way ANOVA; Bonferroni *post hoc* test	$^{*}P < 0.05$
5F	N = 3-4 experiments	Two-way ANOVA; Bonferroni *post hoc* test	$^{*}P < 0.05$; $^{***}P < 0.001$; $^{****}P < 0.0001$
6B	N= 3 experiments	Unpaired two-tailed *t* Student	$^{**}P < 0.01$
6C	N = 3 experiments; 25-33 neurons/group	Two-way ANOVA; Bonferroni *post hoc* test	$^{**}P < 0.01$
6D	N = 4 experiments	Unpaired two-tailed *t* Student	$P = 0.0525$
6E	N = 3 experiments; 44-56 neurons/group	Two-way ANOVA; Bonferroni *post hoc* test	$^{*}P < 0.05$; $^{**}P < 0.01$
Fig1-figSup1A	N = 4 experiments	One-way ANOVA; Bonferroni *post hoc* test	$^{*}P < 0.05$, $^{**}P < 0.01$, $^{***}P < 0.001$

Fig1-figSup1B	N = 3 experiments	One-way ANOVA; Bonferroni *post hoc* test	**P* < 0.05
Fig3-figSup1A	N = 3 experiments; 26-33 neurons/group	Two-way ANOVA; Bonferroni *post hoc* test	**P* < 0.05, ***P* < 0.01, *****P* < 0.0001
Fig6-figSup1	N = 3 experiments; 26-33 neurons/group	Two-way ANOVA; Bonferroni *post hoc* test	***P* < 0.0
Fig6-figSup2	N = 3 experiments; 24-41 neurons/group	Two-way ANOVA; Bonferroni *post hoc* test	****P* < 0.001, *****P* < 0.0001

eLife Sciences Publications, Ltd is a limited liability non-profit non-stock corporation incorporated in the State of Delaware, USA, with company number 5030732, and is registered in the UK with company number FC030576 and branch number BR015634 at the address 1st Floor, 24 Hills Road, Cambridge CB2 1JP | August 2014
